# Gastric Trichobezoar in a 6-Year Old Girl

**Published:** 2015-01-01

**Authors:** NM Joshi, RS Shah

**Affiliations:** 1 Department of General Surgery, P.D. Hinduja Hospital and MRC. VS Marg, Mahim, Mumbai – 400020.; 2 Department of Paediatric Surgery, P.D. Hinduja Hospital and MRC. VS Marg, Mahim, Mumbai – 400020.

**Dear Sir,**

More than 90% trichobezoar are found in young girls between 15 and 20 year of age. Rarely trichobezoar are reported in young girls. About 10% of patients show psychiatric abnormalities termed as trichotillomania or mental retardation.[1]

A 6-year old girl was seen by local practitioner with complaints of fever. On routine examination a lump was palpable in the epigastric and the left hypochondriac regions. There were no associated symptoms like hematemesis, melena, abdominal pain, distension of abdomen or vomiting. There was no history of abnormal behavior. Her developmental history was appropriate for age. The patient had not gained any weight over the last 1 year. There was no history of trichophagia. On examination of abdomen, it was soft, non-distended, with non-tender firm lump palpable in the epigastrium and left hypochondrium. The lump was barely mobile. The patient was anemic, which was treated by oral iron supplements. An ultrasound examination of the abdomen showed echogenic opacities in stomach suggestive of food particles. The CT scan of abdomen showed presence of a well circumscribed mass in the body and pylorus of stomach showing typical whorled appearance suggestive of trichobezoar. The upper GI endoscopy confirmed the presence of fairly large size of trichobezoar in the stomach without any extension to the duodenum (Fig.1). Laparotomy was performed and trichobezoar was manipulated through the gastrotomy incision and removed in one piece (Fig.2). Post-operative period was uneventful. A psychiatry opinion was sought during the hospital stay which was normal. The child was advised for follow-up.

**Figure:**
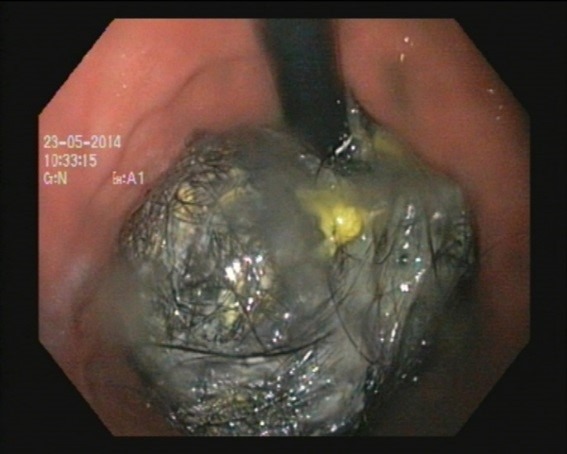
Figure 1:Trichobezoar as seen during endoscopy.

**Figure:**
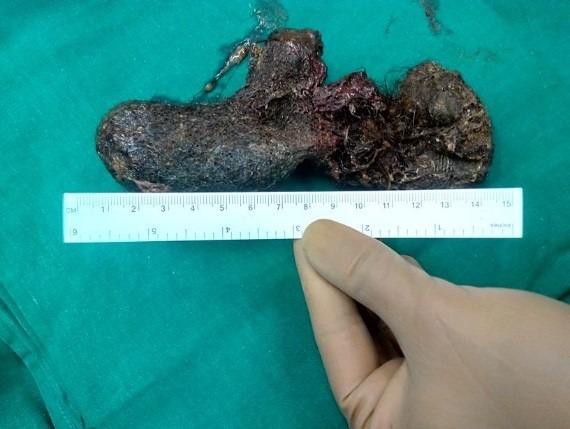
Figure 2: Retrieved gastric trichobezoar.

The patients can present with malnutrition, weight loss, and abdominal pain with signs of GI obstruction or perforation.[2] Early onset hair pulling (before 8 years) is benign and self-limited.[3] Our patient presented with asymptomatic abdominal lump, failure to gain weight and anemia. It was astonishing that how and why parents remained unaware of trichophagia for such a long period that the child developed a big trichobezoar. Although not a therapeutic option, endoscopy is extremely valuable as a diagnostic modality in patients in whom the nature of the gastric mass is unclear. It enables the differentiation between trichobezoar and foreign bodies that can be fragmented and removed using endoscopy.[4] Even in our case, the patient had upper GI endoscopy for diagnostic and therapeutic purposes. But decision to perform a laparotomy was taken due to large size of trichobezoar. Many of these patients have psychiatric pathology with emotional problems, family discord, and history of neglect or mental retardation. Counselling by a psychiatrist is an important part of management to prevent recurrence.[5] In our case although the psychiatric evaluation was normal, but concealing the history of trichophagia by the patient himself may point to some underlying psychological or psychiatric problem.

## Footnotes

**Source of Support:** Nil

**Conflict of Interest:** None declared

